# Cysteine Redox Chemistry in Peptide Self-Assembly to Modulate Hydrogelation

**DOI:** 10.3390/molecules28134970

**Published:** 2023-06-24

**Authors:** Maria Cristina Cringoli, Silvia Marchesan

**Affiliations:** Department of Chemical and Pharmaceutical Sciences, University of Trieste, 34127 Trieste, Italy

**Keywords:** cysteine, peptide, self-assembly, disulfide, thiol, redox, hydrogels, supramolecular, fibrils, amyloid

## Abstract

Cysteine redox chemistry is widely used in nature to direct protein assembly, and in recent years it has inspired chemists to design self-assembling peptides too. In this concise review, we describe the progress in the field focusing on the recent advancements that make use of Cys thiol–disulfide redox chemistry to modulate hydrogelation of various peptide classes.

## 1. Introduction

Nature makes wide use of cysteine (Cys) thiol reactivity to direct protein assembly into functional structures [1], especially through its oxidation to disulfide bridges [2]. Other types of thiol reactivity in naturally occurring systems include metal coordination, for instance in metalloproteins, also towards catalytic function [3,4]. Moreover, sulfur metabolism deriving from Cys (and methionine, Met) sustains the redox chemistry that serves as the cellular antioxidant system. It also mediates signaling within and between cells [5,6,7,8]. The plethora of Cys roles in functional proteins and their complexes is too vast to provide a comprehensive list here; however, it is worth mentioning a few key examples that are crucial in the biochemistry of living organisms.

Cys thiol oxidation into disulfide bridges is a key step of protein folding that proceeds especially in the lumen of the endoplasmic reticulum (ER). It is subjected to a quality control system before proteins can leave the ER, often to be shuttled to the cell surface [9]. There, they are exposed to harsher conditions, and disulfide bridges can exert an important stabilizing role to preserve their function [10]. Important examples include:

- Hormones, such as insulin, whose function is strongly dependent on correct disulfide formation [11,12];
- Functional proteins of the immune system, such as antigen-presenting major histocompatibility complexes (MHCs) [13] and antibodies [14];
- Natural antimicrobial peptides [15], such as defensins [16,17];
- Respiratory complexes that are key for cell metabolism, such as cytochrome c [18];
- Proteins of the extracellular matrix, such as collagen [19];
- Focal adhesion complexes that link integrins to the cytoskeleton in key processes, such as cell adhesion and migration [20,21];
- Several toxins and venom peptides [22,23,24];
- Ubiquitin transfer between catalytic cysteines leading to protein degradation [25,26];
- Enzymes controlling transduction pathways, such as phospodiesterases [27].

**Figure 1:**
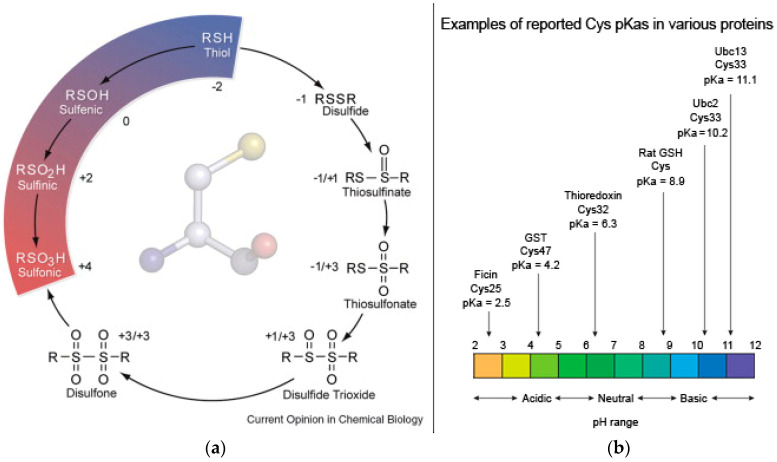
(**a**) Cys oxidation products that can occur through protein post-translational modification, with sulfur oxidation numbers shown next to each species. Reproduced from [30], Copyright © 2023, with permission from Elsevier. (**b**) Examples of reported pKa values for Cys in various proteins over the pH range 2.5–11.1. Values extracted from [32] and works cited therein.

Controlling the correct formation of disulfide bonds to produce functional proteins in vitro is not trivial. Several approaches have been developed to master oxidative folding of recombinant proteins and peptides [28,29]. It is thus not surprising that bioinspired approaches that aim to exploit thiol oxidation to disulfides to control peptide assembly have appeared in the literature in the past in relatively modest numbers. This observation is particularly true if we also consider the requirement to attain hydrogels, since crosslinking can result in precipitation instead. Furthermore, Cys oxidation can proceed further to oxygen-containing groups, such as sulfenic (RSOH), sulfinic (RSO_2_H), and sulfonic (RSO_3_H) acids (Figure 1a), which have been observed in proteins [30]. However, the most common oxidation product is the disulfide, which can be readily formed at pH values higher than the pKa of Cys, thanks to the nucleophilicity of the thiolate anion. Cys has an intrinsic pKa of 8.6, which can vary depending on its position in a peptide or protein sequence, and which can be calculated by several methods [31]. The resulting variability in pKa values is high (Figure 1b), reaching values as little as 2.5 and as high as 11.1 in catalytic active sites [32]. Modulation of Cys thiol pKa is indeed an interesting strategy to promote disulfide crosslink formation even at physiological pH values, to yield hydrogels for biological uses [33]. In recent years, Cys thiol oxidation to disulfide has been increasingly and successfully applied as a convenient trigger to modulate hydrogels obtained from peptides and proteins, as described further below.

## 2. Cys Thiol–Disulfide Redox Chemistry to Modulate Peptide Hydrogels

### 2.1. β-Sheet Peptides for Hydrogels

β-Sheet peptides have become popular building blocks to attain hydrogels. Numerous natural amyloids form hydrogels based on β-sheets and cross β-structures [34]. A typical design of β-sheet-forming peptide hydrogelators features alternating hydrophilic and hydrophobic amino acids, so as to create a polar and an apolar surface on the two opposing sides of the β-sheets. In this manner, through self-assembly, nanofibrils can arise that entangle in hydrogel matrices [35,36]. This approach, which originally featured long peptides [37,38], has also been successfully applied to gradually shorter sequences, which have the advantage of lower costs and easier preparation [39,40,41,42,43,44]. Alternatively, inclusion of D-amino acids into heterochiral sequences can yield hydrogels from hydrophobic amino acids, so that the polar surface is composed of the peptide backbone, and the apolar surface is composed of the sidechains [45,46,47]. In this manner, peptide sequences as short as two amino acids provided hydrogels with good cytocompatibility in vitro [48,49]. Lastly, the use of aromatic N-caps has provided a plethora of hydrogelators from amino acids and short peptides [50,51,52].

**Figure 2:**
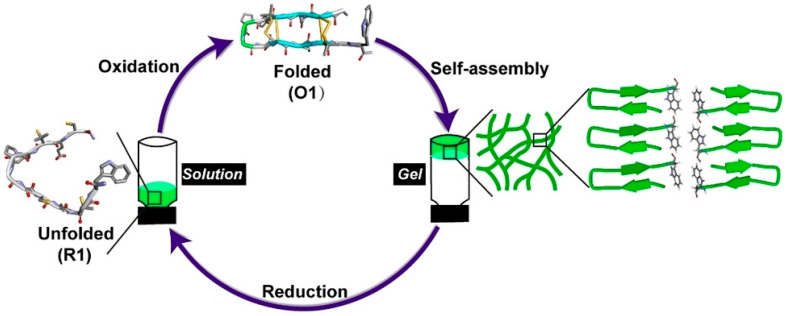
Schematic representation of a redox-responsive hydrogel that forms upon oxidation of a random-coil peptide to yield a self-assembling β-hairpin, while gel-to-sol transition is triggered by disulfide reduction. Reproduced with permission from [54], © 2023 Wiley-VCH GmbH.

However, the application of Cys redox chemistry to crosslink β-sheet hydrogelators has been reported mainly in recent years. Lanreotide, a heterochiral peptide, forms nanotubes that gel and that are based on a β-hairpin that is stabilized by an intramolecular disulfide bridge [53]. Recently, a thiol-rich peptide comprising Cys and penicillamine residues was demonstrated to form hetero-disulfide bonds to yield amphipathic β-hairpins that gel (Figure 2). In the presence of a reductant, such as dithiothreitol, the disulfide bridges are removed, and the consequent conformational switch to a random coil triggers the gel-to-sol transition [54]. Another recent study showed that the sol-to-gel transition could be triggered for an antimicrobial cationic heptapeptide featuring a C-terminal Cys. In this case, gelation occurred upon pH increase to induce disulfide-bond-mediated dimerization, leading to the formation of β-sheets [55]. Using a similar approach, Fmoc-Phe-Phe-Cys dimerization was exploited as a means to convert worm-like micelles into coiled nanohelices that yielded a printable hydrogel [56]. The tetrapeptides Ac-Val-Val-Lys-Cys-NH_2_ and Ac-Phe-Phe-Lys-Cys-NH_2_ provide another couple of examples where C-terminal Cys dimerization via disulfide-bridge crosslinking yielded thixotropic and injectable hydrogels. These soft materials were envisaged for biomaterial applications, thanks also to their responsive behavior to glutathione levels [57]. Hauser and collaborators recently described the tetrapeptide Ac-Ile-Val-Lys-Cys that formed hydrogels, whose stiffness was dramatically increased upon dimerization via disulfide crosslinks in the presence of hydrogen peroxide as an oxidizing agent [58].

Nevertheless, it is worth noting that, despite all these success stories, the mere introduction of Cys amino acids into self-assembling peptide sequences can affect the supramolecular and viscoelastic behavior in ways that are not always easy to predict. Indeed, both the number and the position of Cys residues are important factors in determining such effects on the resulting assemblies, and on their ability to form macroscopic gels, as recently described for the amphipathic sequence EAK16-II [59]. Often, introduction of a Cys residue at the C-terminal position offers a safe approach to avoid the disruption of the assemblies and yields end-to-end crosslinking. This concept has been demonstrated on amyloid proteins such as α-synuclein which yielded self-healing hydrogels and aerogels [60].

**Figure 3:**
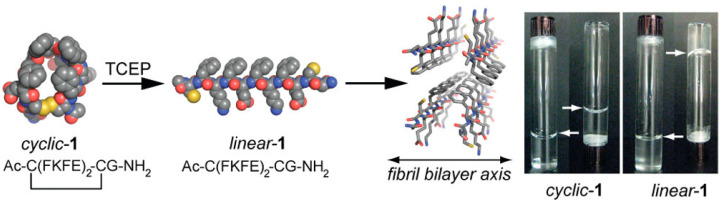
Cyclization of a peptide through a disulfide bond induces a conformational restraint that prevents hydrogelation, while a reductive trigger produces the linear peptide that gels. Reproduced with permission from [61]. Copyright © 2023, American Chemical Society.

The establishment of disulfide bonds does not always enable hydrogelation from otherwise soluble peptides. The opposite effect can also be attained upon appropriate design. For example, disulfide bonds had been previously used to cyclize an amphiphilic peptide to provide a conformational restraint that prevented hydrogelation and maintained the peptide in solution. In this case, it was the reduction of the disulfide form to Cys thiols that produced the linear peptide molecules, which could assemble into a hydrogel based on β-sheets [61], as shown schematically in Figure 3.

Dodero and collaborators have recently reported a redox mechanism to modulate amyloid fibrillation of a Cys-containing tripeptide, namely Phe-Phe-Cys, which was acetylated at the N-terminus and amidated at the C-terminus. The supramolecular behavior of this sequence was determined by the oxidation state of the Cys sidechain thiol group. In particular, in reductive environments, 60 nm wide nanospheres were formed upon application of a solvent switch. In contrast, oxidative conditions at the alkaline pH of 8 formed the disulfide-bound dimer that further self-assembled into nanofibrils with a 20 nm diameter. Furthermore, the conversion was reversible upon the addition of a reducing agent. Although no hydrogel was reported in this case, this study provided an elegant example of a nanomorphological switch based on Cys redox chemistry of a minimalistic sequence as a simple as a tripeptide [62] that exploited the amyloid-derived Phe-Phe self-assembling motif [63].

Another minimalistic system that exploited Cys redox chemistry was recently reported by Pramanik and collaborators. In this work, an azobenzene moiety was bound to the dipeptide Lys-Cys, so that oxidation of the C-terminal thiol to disulfide could yield a photoresponsive, thixotropic, and injectable hydrogel that was envisaged for dye removal from contaminated waters [64]. Inclusion of azobenzene terminal moieties was successfully applied also to the oxidized form of glutathione, to yield smart hydrogels that could respond to a variety of stimuli [65]. Finally, Diaferia et al. reported the case of a heptapeptide hydrogelator that, upon oxidation of the Cys residue central to the sequence, yielded a hydrogel with significantly enhanced rigidity, thus offering the possibility to modulate the viscoelastic properties of the material, depending on the Cys redox chemistry [66]. Finally, Banerji and co-workers reported a superhydrogelator featuring a cyclodipeptide based on Leu and Cys that was S-protected with a benzyl moiety. The thermoresponsive system successfully co-assembled in the presence of the antitumoral drug 5-fluoruracil for its sustained release, and it demonstrated a remarkable stability over the wide pH range from 6 to 12. The thermoresponsiveness was determined by the breaking of the intermolecular H-bonding network between amide groups induced by heating, and its re-establishment upon subsequent cooling [67]. This result is a useful advancement in the field, considering that the hydrophilic drug was released very rapidly in other cases when co-assembled with short-peptide molecules used for the same purpose [68].

### 2.2. Peptide Amphiphiles

Peptide amphiphiles are another popular class of hydrogelators, whereby an alkyl chain is bound to the peptide sequence typically through amidation with a fatty acid [69]. They have become a popular class of building blocks used as scaffolds for tissue engineering [70]. It is thus not too surprising that the chemical conversion of Cys thiol groups to disulfides and vice versa has also been applied to this class of hydrogelators to modulate their viscoelastic properties and supramolecular behavior. In particular, the use of chemically modified poly (Cys) chains featuring disulfide bridges yielded self-assembling amphiphiles that formed micelles or nanofibrous hydrogels based on β-sheets at the physiological pH 7.4 [71]. These systems have been envisaged for the loading and release of bioactive compounds, such as drugs.

In another example, the dipeptide sequence Lys-Cys was conjugated at the N-terminus to a pyrene unit with an alkyl chain linker [72]. In this manner, the peptide amphiphile molecules could form dimers through disulfide bridges between their C-termini, to yield stable hydrogels. These soft materials did not dissolve in water and have been envisaged as carriers of proteins to protect them from chemical environments. This type of vehicle could thus offer a promising avenue for the formulation of biotherapeutics that are more sensitive to physico-chemical changes in their surrounding environments, and to ensure longer-term stability.

### 2.3. Polypept(o)ides for Hydrogels

Peptoids have attracted great interest as peptide mimics and as building blocks for hydrogel biomaterials [73]. They consist of poly(*N*-substituted glycine), whereby the variable residues on the nitrogen atom can mimic amino acid sidechains, with the net advantage of added resistance against protease-mediated hydrolysis [74]. They are typically produced by solid-phase methods, but also in liquid phase, and their synthesis is continuously being optimized to the benefit of researchers interested in their use [75]. However, their altered ability to engage in H-bonds, relative to peptide analogs, can lead to a reduced ability to form hydrogels [76]. It is thus not surprising that, often, peptoids are combined with peptides [77], polysaccharides [78,79], or synthetic polymers [80] to stabilize the resulting soft matter. In particular, inclusion of Cys in polysarcosines has been exploited to crosslink polypept(o)ides via disulfide bridges to control the morphology of the resulting micellar assemblies [81].

### 2.4. Coiled Coils and α-Helical Peptides for Hydrogels

Coiled coils feature repeating units that typically comprise seven amino acids that are indicated as *abcdefg*, where usually the *a* and *d* residues are hydrophobic, while the *e* and *g* amino acids are charged. In this manner, the heptad motif folds into amphipathic α-helices, with non-covalent interactions stabilizing their association into parallel clusters that compose the coils, which result from multiple units of the same monomer or of different monomers [82]. These building blocks have been widely applied as biomaterials [83]. They are typically produced as recombinant proteins through biotechnological tools, especially by means of expression in suitable host cells, such as *E. coli* strains, although their chemical synthesis is possible too [84].

**Figure 4:**
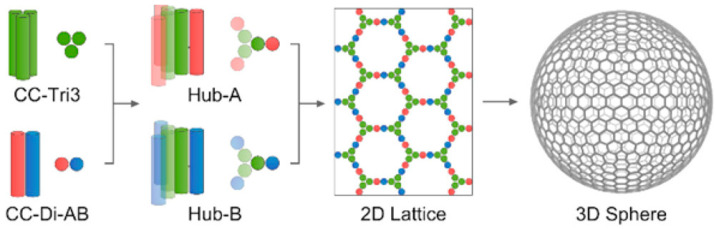
Disulfide bridges between coiled coils CC-Tri3 and CC-DI-AB enable the formation of a hexagonal lattice that constitutes the surface of a self-assembled peptide particle. Adapted with permission from [93]. Copyright © 2023, American Chemical Society.

Hydrogels can also be attained from the hierarchical organization of coiled coils. For instance, Montclare and co-workers designed the protein Q that forms α-helices that assemble into coiled coils that yield thermoresponsive hydrogels [85]. The interior of the coiled coils is hydrophobic and it was exploited for the loading of curcumin as a drug model, and its subsequent sustained release over two weeks was studied. Appropriate design to include charged residues was successfully applied by the same group [86], Dexter and collaborators [87], and by Chmielewski and coworkers [88], to impart pH-responsiveness to coiled-coil hydrogels. Strategic inclusion of Cys units can be exploited to guide hierarchical self-assembly towards hydrogels for tissue engineering, with the possibility to include bioactive motifs. An example includes the fibronectin-derived RGD, to impart adhesiveness to cells [89]. Indeed, the formation of disulfide bridges is a convenient strategy to control the side-by-side association between the coils, through the inclusion of Cys residues into defined positions of the helical peptides [90]. In particular, Woolfson and co-workers have applied this strategy to obtain self-assembling cage-like particles (Figure 4) [91,92,93].

**Figure 5:**
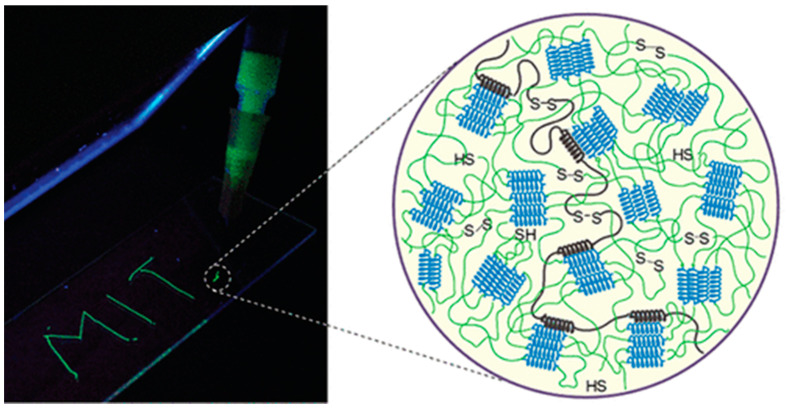
Disulfide bridges between Cys residues can modulate chain extension and entanglement in coiled-coil hydrogels. Reproduced with permission from [94]. Copyright © 2023, American Chemical Society.

Alternatively, Cys thiol oxidation can be exploited to control protein chain extension and entanglement by including these residues near the N- and C-termini (Figure 5) to ameliorate the mechanical properties of the resulting hydrogels, as demonstrated by Olsen and coworkers [94].

This type of end-to-end crosslinking was also successfully applied to obtain collagen-like peptide polymers that displayed the additional feature of presenting bioactive motifs capable of integrin recognition. They could thus be used for platelet adhesion and activation, for instance, towards thrombotic activity and wound healing [95]. Disulfide engineering has also been applied to recombinant collagen-like proteins to yield tunable hydrogels with promising biocompatibility [96]. Finally, α-helical keratin also inspired the use of Cys thiol–disulfide redox chemistry to modulate the viscoelastic properties of the resulting hydrogels. Interestingly, upon oxidation of Cys thiol groups to disulfides, the α-helical content significantly decreased in favor of β-sheet conformations. The resulting soft matter demonstrated injectability, a self-healing ability, and good cytocompatibility with fibroblast cells in vitro [97]. Finally, disulfide crosslinks have also been recently applied to attain hydrogels from buckwheat protein lysates that featured varying secondary structures and viscoelastic properties, depending on the applied experimental conditions [98].

## 3. Conclusions

In conclusion, over the last decade, we have witnessed the increasing application of Cys thiol–disulfide redox chemistry to modulate the viscoelastic behavior of supramolecular peptide-based systems (see summary Table 1). The vast majority have been envisaged for biological use, but also as green materials for environmental remediation. Many of these systems exploit the formation of disulfide crosslinks to induce gelation. The opposite is more rare, although it has been applied, for instance, by exploiting the disulfide bond as a means to impart a conformational constraint that impedes gelation. In other examples discussed above, disulfide bridges enable an increase in the hydrogel stiffness, thus offering a tool to modulate the viscoelastic properties of the soft matter, depending on the intended use. Many of these studies took inspiration from nature’s strategic use of Cys redox chemistry, and we can envisage that this is just the beginning for the generation of even more versatile applications of design strategies that are bioinspired. For instance, it was found that more than 10% of disulfides that are present in the Protein Data Bank are strained, and thus more reactive; a canonical case occurs between antiparallel β-strands that are destabilized due to alteration in their hydrogen-bonding pattern [99]. It is reasonable to think that this type of increased reactivity could be exploited to impart catalytic activity to a supramolecular system, and in the gel phase. Other forms of supramolecular assemblies could be catalytically active, especially through the mimicry of the protected environment of enzyme hydrophobic pockets for reactions to occur. For instance, a cyclic dipeptide featuring Cys was already demonstrated to display an esterase-mimicry ability when co-assembled with another cyclic dipeptide featuring His, which is a recurrent catalytically active amino acid [100]. Cyclodipeptides are indeed emerging as industrially attractive building blocks for hydrogels [101], which can be cost effective, biocompatible, and biodegradable. Furthermore, this class of biomolecules, despite their chemical simplicity, can display unexpected bioactivity, such as anti-ageing effects [102,103].

Medicine is certainly a field where this type of materials can find various applications to improve human health. As building blocks, peptides are well-positioned in terms of biocompatibility by design, and they can also be produced by green methods and biotechnological tools to preserve the environment. In particular, responsive formulations for sustained or ad hoc release of drugs and biotherapeutics are very appealing. For example, disulfide-linked prodrugs have been successfully applied towards on-demand drug release [104]. Furthermore, a cystine-linked peptide co-assembled with curcumin as an antitumoral drug model enabled its release in the presence of glutathione, which is overexpressed in tumor microenvironments [105]. This type of approach is thus particularly promising in cancer therapy to minimize chemotherapeutics’ side effects and enable their targeted delivery and, thus, the use of lower amounts of active principle ingredients. Furthermore, Cys as an amino acid was recently reported for its ability to disrupt amyloid formation in a minimalistic model in vitro [106]. This finding gives scope for further applications to modulate the bioactivity of Cys-containing hydrogels, and to potentially design amyloid fibrillation inhibitors. In this manner, innovative therapeutic solutions could be developed in the area of neurodegeneration and beyond.

From a fundamental science point of view, we are witnessing great advances in supramolecular chemistry, with great efforts worldwide to translate the progress made in recent decades in molecular machines, into innovative technologies. In this regard, the interlocking of molecules plays a central role. Interestingly, Cys thiol oxidation has also been recently applied to attain mechanically interlocked peptides [107], and to obtain catenanes from lasso peptides [108], thus adding a further level of topological complexity to peptide-based supramolecular systems. To conclude, this is just the beginning of the successful application of Cys redox chemistry to design responsive supramolecular hydrogels and to tune their viscoelastic properties. It is thus envisaged that these studies advancing the knowledge in the field provide a solid foundation to enable further progress in various applications for peptide-based hydrogels.

## Figures and Tables

**Table 1:** Modulation of hydrogelation using Cys redox chemistry from the examples discussed in this review.

Peptide Sequence	Reduced Cys	Oxidized Cys	Ref.
GCEPenYPGSCKPenGW ^1^	Sol	Gel	[54]
GCEPenYPGSCKPenG ^1^	Sol	Ppt ^2^	[54]
WGCEPenYPGSCKPenGW ^1^	Sol	Ppt ^2^	[54]
GCEVYPGSAKPenGW ^1^	Sol	Sol	[54]
GAEPenYPGSCKVGW ^1^	Sol	Sol	[54]
Ac-RKKWFWC-NH_2_	Sol	Gel	[55]
Fmoc-FFC	Sol	Gel	[56]
GGKC-NH_2_	Sol	Sol	[57]
AAKC-NH_2_	Sol	Sol	[57]
IIKC-NH_2_	Sol	Sol	[57]
LLKC-NH_2_	Sol	Sol	[57]
VVKC-NH_2_	Sol	Sol	[57]
FFKC-NH_2_	Sol	Sol	[57]
Ac-GGKC-NH_2_	Sol	Sol	[57]
Ac-AAKC-NH_2_	Sol	Sol	[57]
Ac-IIKC-NH_2_	Ppt ^2^	Ppt ^2^	[57]
Ac-LLKC-NH_2_	Sol	Sol	[57]
Ac-VVKC-NH_2_	Sol	Gel	[57]
Ac-FFKC-NH_2_	Sol	Gel	[57]
Ac-IVKC	Sol	Gel	[58]
CAEAEAKAKAEAEAKAK-NH_2_	Gel	Gel	[59]
CAEAEAKAKAEAEAKAKC-NH_2_	Gel	Gel	[59]
α-synuclein (Y136C)	Sol	Gel	[60]
Ac-CFKFEFKFECG-NH_2_	Gel	Sol	[61]
Azo-KC-NH_2_^3^	Sol	Gel	[64]
*Azo*-GSH dimethyl ester ^4^	Sol	Gel	[65]
FYFCFYF-NH_2_	Gel	Gel	[66]
hexyl-poly(Cys-SS-CH_2_CH_2_COOH)	Sol	Gel	[71]
dodecyl-poly(Cys-SS-CH_2_CH_2_COOH)	Sol	Gel	[71]
octadecyl-poly(Cys-SS-CH_2_CH_2_COOH)	Sol	Gel	[71]
4-(pyren-1-yl)butanoyl-KC-NH_2_	Sol	Gel	[72]
4-(pyren-1-yl)butanoyl-KC	Sol	Gel	[72]
4-(pyren-1-yl)butanoyl-kC	Sol	Gel	[72]
4-(pyren-1-yl)butanoyl-Kc	Sol	Gel	[72]
Collagen-like peptides	Sol	Gel	[95,96]
Keratins	Sol	Gel	[97]
Buckwheat peptides	Sol	Gel	[98]

^1^ Pen = penicillamine. ^2^ Ppt = precipitate. ^3^ Azo = 4-(4-(phenyldiazenyl)phenoxy)butanoyl. ^4^ Azo = 4-(4-(phenyldiazenyl)phenoxy)pentanoyl and GSH = glutathione.

## Data Availability

Not applicable.

## Sample Availability

Not available.
